# Supplementary figures and images for: Fruitless decommissions regulatory elements to implement cell-type-specific neuronal masculinization
Source: PLoS Genet. 2021 Feb 18;17(2):e1009338. doi: 10.1371/journal.pgen.1009338 (PMC7924761; doi:10.1371/journal.pgen.1009338)

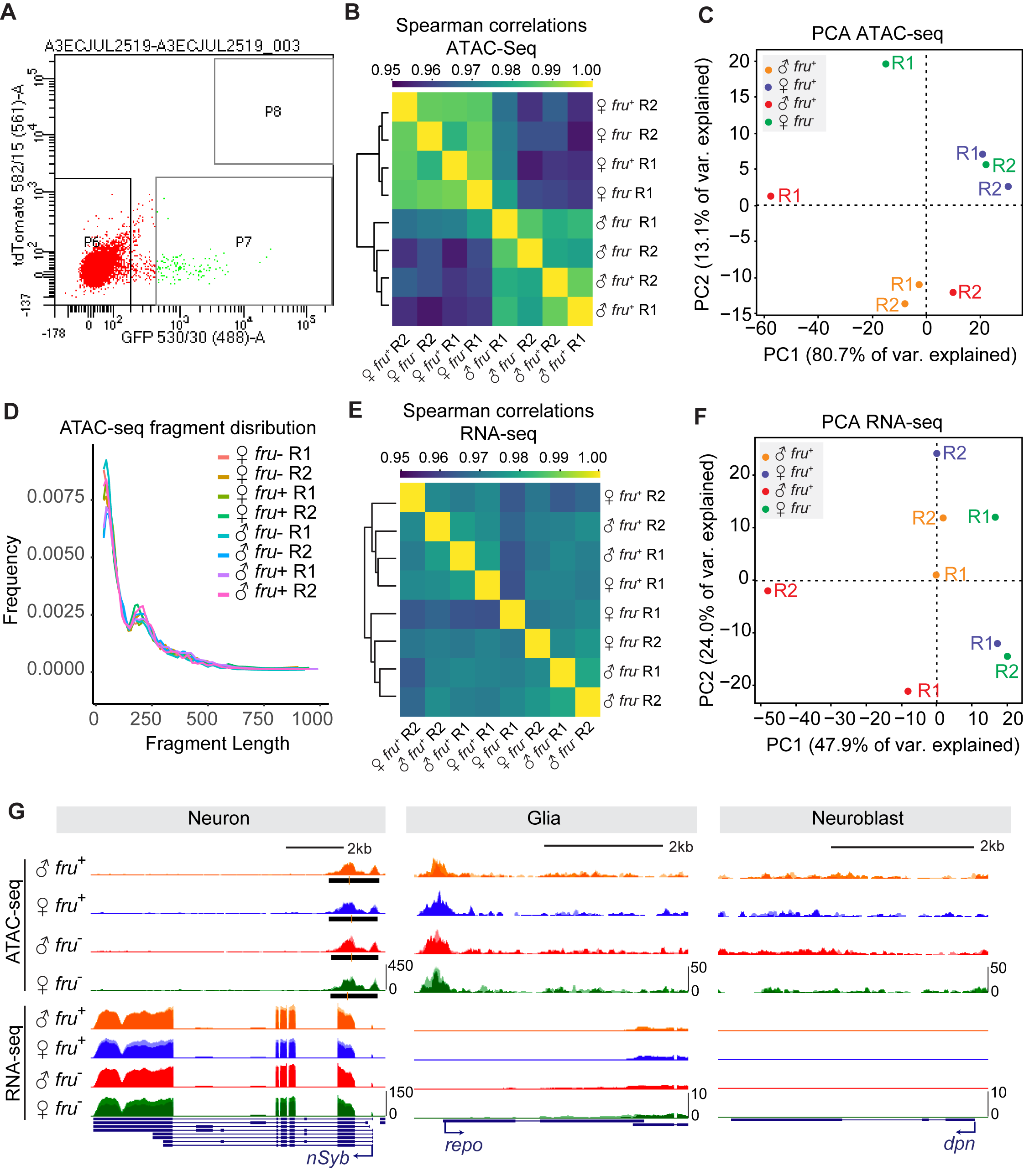

Supplement: S1 Fig — A. Example FACS plot showing gates for fru+ (GFP+) neurons collected. Example plot corresponds to male neurons from replicate 2 of RNA-seq experiments. B. Heatmap of Spearman correlations of uniquely aligned, deduplicated reads from ATAC-seq libraries. C. PCA analysis of uniquely aligned, deduplicated reads from ATAC-seq libraries. PC1 reflects read depth. D. Fragment length of ATAC-seq libraries. E. Heatmap of Spearman correlations of uniquely aligned, deduplicated reads from RNA-seq libraries. F. PCA analysis of uniquely aligned, deduplicated reads from RNA-seq libraries. G. UCSC genome browser screenshots of ATAC-seq and RNA-seq signal across neural (nSyb), glial (repo), and neuroblast (dpn) specific genes. (TIF) [file pgen.1009338.s001.tif]

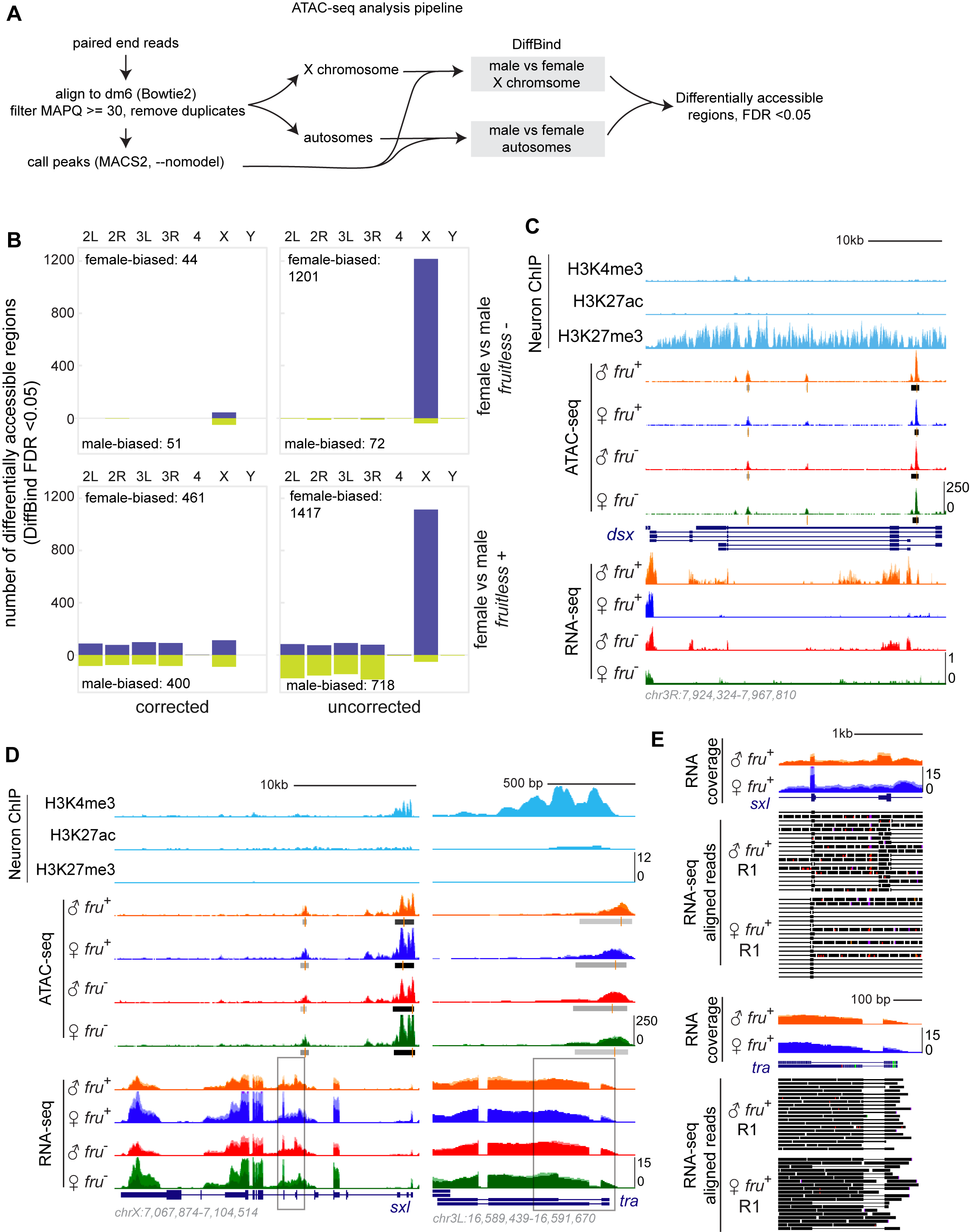

Supplement: S2 Fig — A. Flowchart of computational pipeline used to identify differentially accessible regions between male and female samples using ATAC-seq. B. Barplots displaying number of differentially accessible regions using DiffBind FDR <0.05 as a cutoff. Corrected regions are produced by the pipeline in (A), while uncorrected regions are produced without splitting of the X chromosome and autosomes before running DiffBind. Colors correspond to Fig 2C, where purple are regions that have female-biased accessibility, and chartreuse represents regions with male biased accessibility. C. UCSC genome browser screenshot of ATAC-seq and RNA-seq signal in the dsx locus. Blue signal tracks represent INTACT histone ChIP data for R57C01(Nsyb)-labeled neurons in the adult head [47]. D. UCSC genome browser screenshots of the sxl and tra loci. E. Zoom in of highlighted regions in D, corresponding to sexually dimorphic splicing events. Displayed are RNA-seq coverage tracks and 20 aligned reads in male fru+ neurons and female fru+ neurons. Reads correspond to replicate 1 data. For both genes, male mRNA contains only non-functional isoforms, while female mRNA contains both protein-coding and non-functional isoforms. (TIF) [file pgen.1009338.s002.tif]

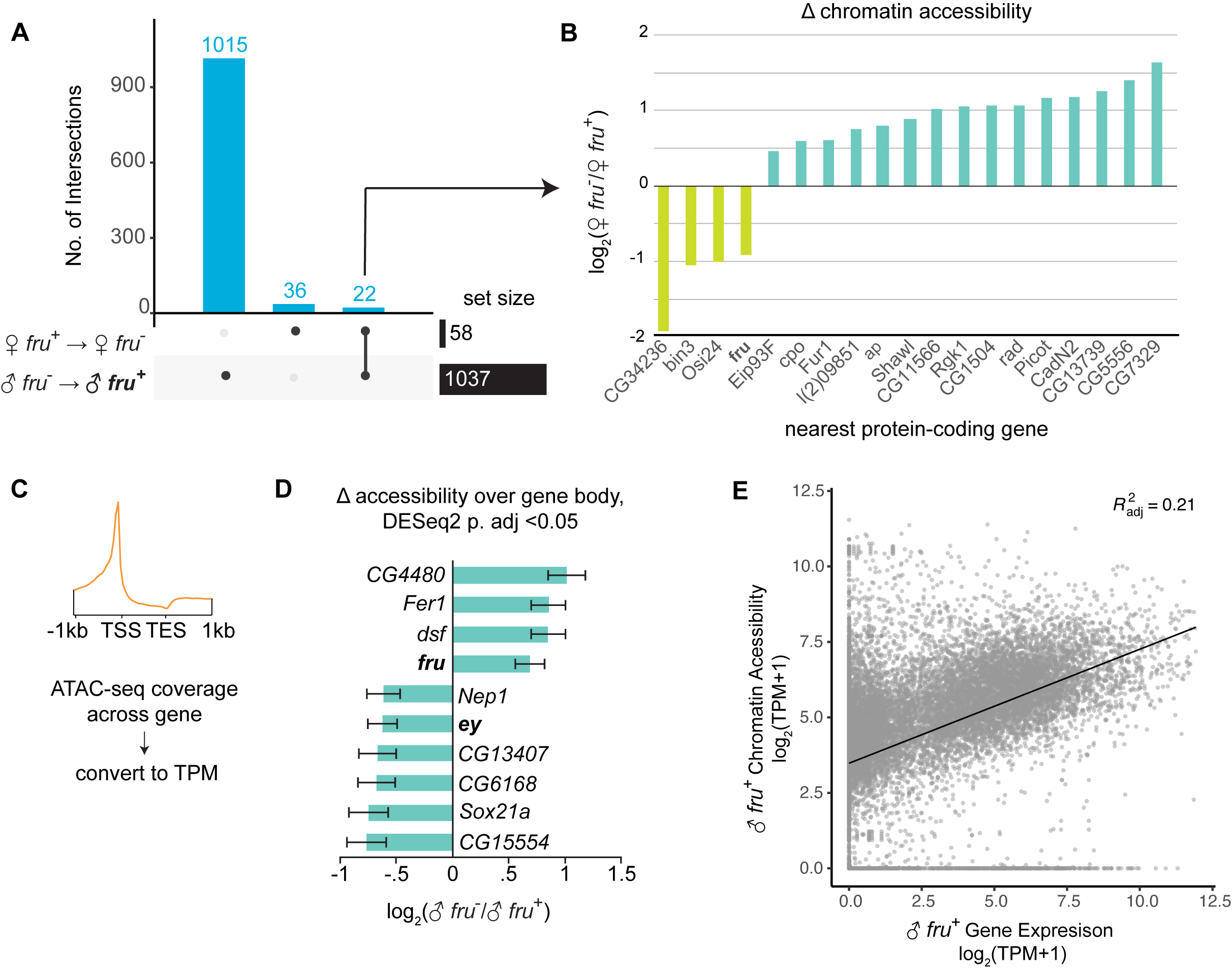

Supplement: S3 Fig — A. UpSet plot showing the intersection of regions which are differentially accessible (DiffBind FDR <0.05) between fru+ and fru- neurons in both sexes. B. Of 22 regions differentially accessible between fru+ and fru- neurons, 19 correspond to protein-coding genes (labeled on the X axis). The log2(Fold Change) in accessibility of these genes relative in female neurons is plotted. C. Schematic of calculating chromatin accessibility across a gene locus. D. Barplot of log2(Fold Change) of genes with gene-scale differential coverage between male fru+ neurons and male fru- neurons. ey is a Kenyon cell marker. E. Scatterplot of whole-gene chromatin accessibility versus gene expression level in the male fru+ dataset. (TIF) [file pgen.1009338.s003.tif]

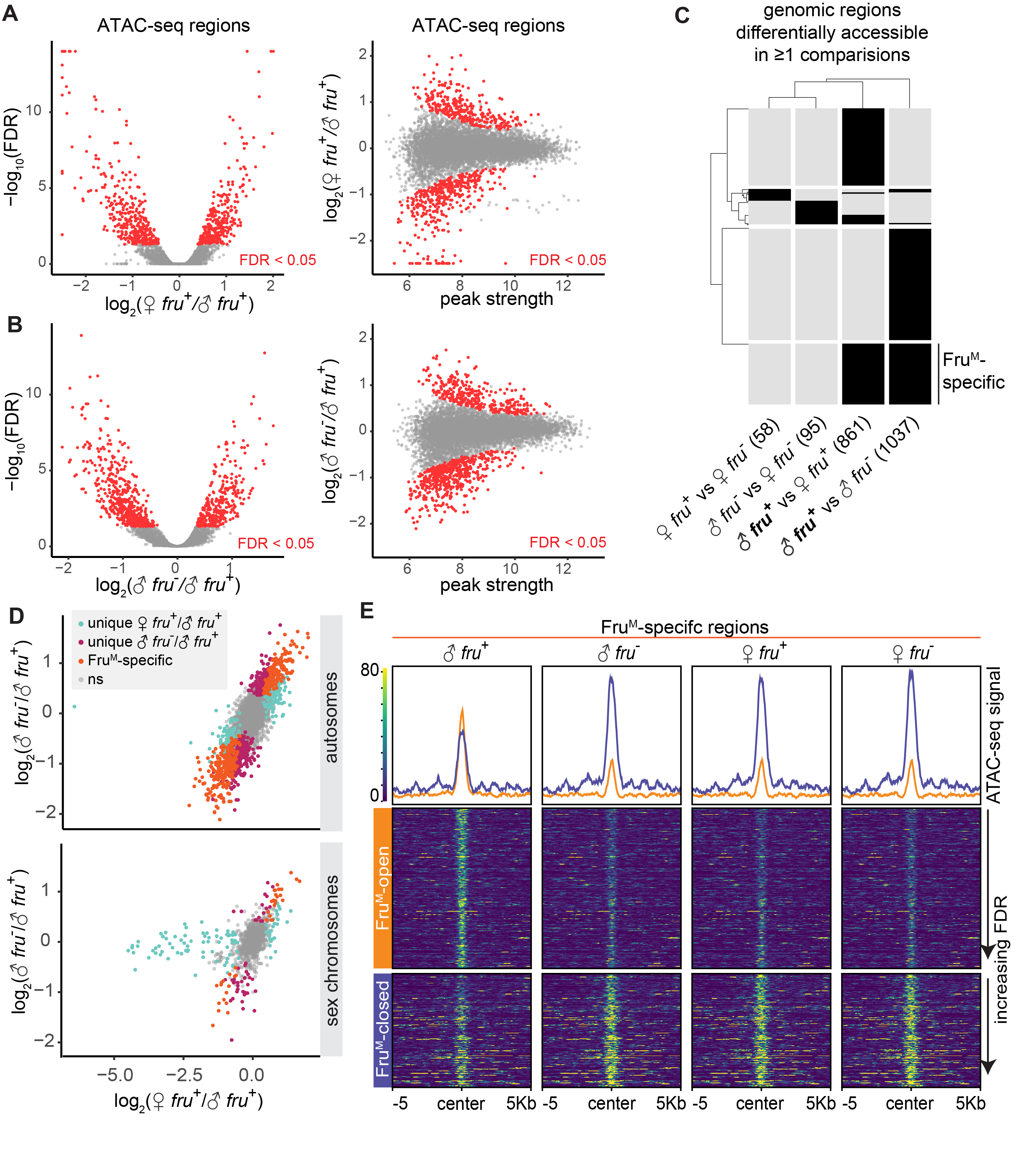

Supplement: S4 Fig — A. Volcano and MA plots of regions with differential accessibility (DiffBind < 0.05) between female and male fru+ neurons. Points at the top border in the volcano plot and along the bottom border in the MA plot have been thresholded such that they are visually comparable to plots in B. B. Volcano and MA plots of regions with differential accessibility (DiffBind < 0.05) between male fru- and fru+ neurons. C. Binary heatmap of regions called differentially accessible in 1 or more comparisons. D. Scatterplot of log2 fold changes in accessibility compared to male fru+ neurons. Figure shows analysis in Fig 4D with autosomal regions (chromosomes 2, 3, and 4) separated from sex chromosomal regions (X and Y). E. Signal heatmaps of FruM-specific regions, separated by regions which are selectively open or closed in male fru+ neurons. (TIF) [file pgen.1009338.s004.tif]

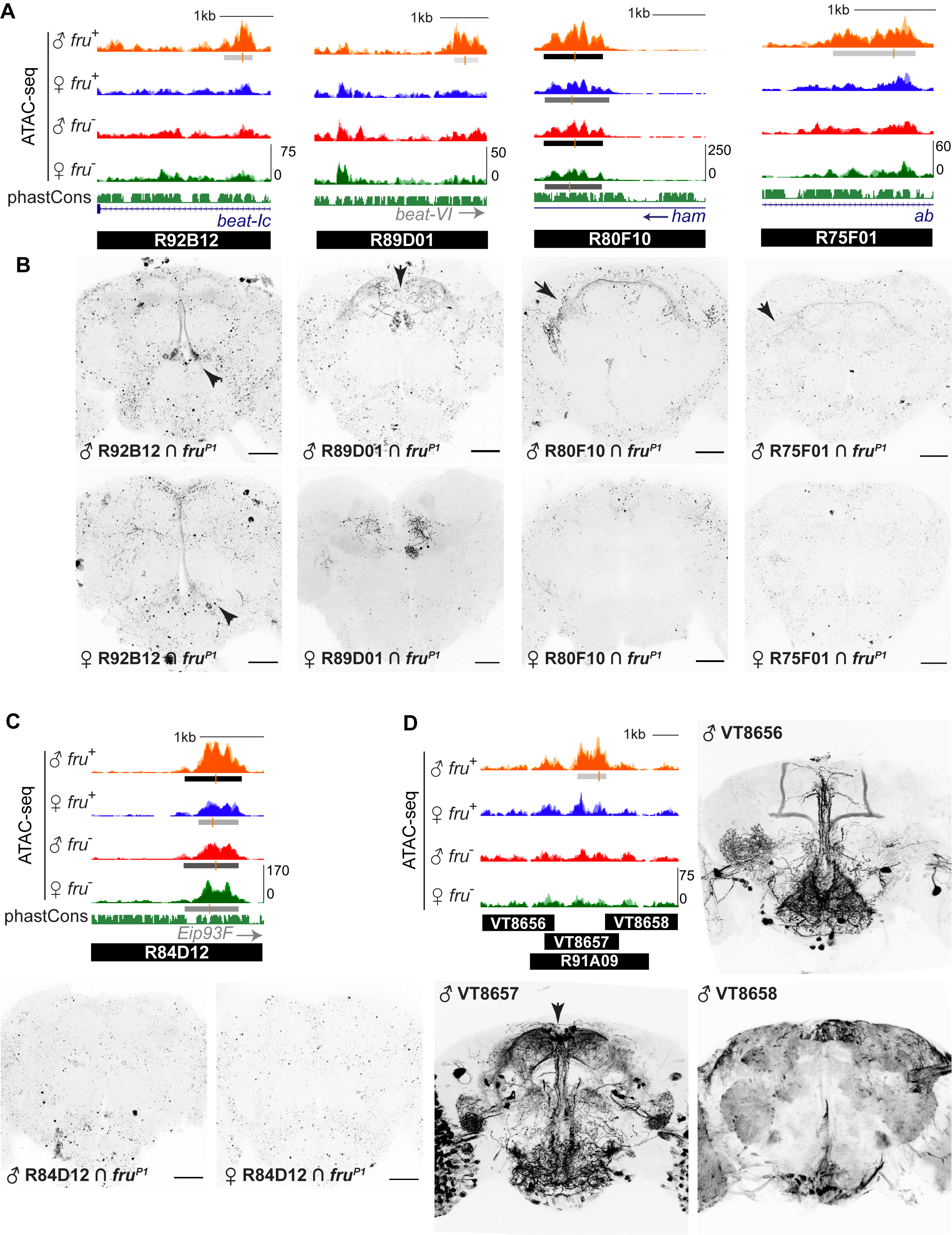

Supplement: S5 Fig — A. UCSC genome browser screenshots of genomic regions covered by each reporter. B. 2-photon stack of adult male (B) and female brains. Arrows point to cells with sex-specific expression (89D01, 80F10, 75F01) or dimorphic labeling intensity (92B12). C. UCSC genome browser screenshot of region covering enhancer reporter 84D12 and 2-photon stack of adult male and female brains. Labeling is mutually exclusive with fru expression. Brain-wide speckle signal is autofluorescence. D. In-silico enhancer bashing of enhancer reporter element 91A09 (Fig 5D–5F). UCSC genome browser screenshot showing overlapping enhancer reporters from the Vienna Tiles collection. Maximum intensity projection of matching substacks for each overlapping tiles from Brainbase. Images correspond to adult male brains. (TIF) [file pgen.1009338.s005.tif]

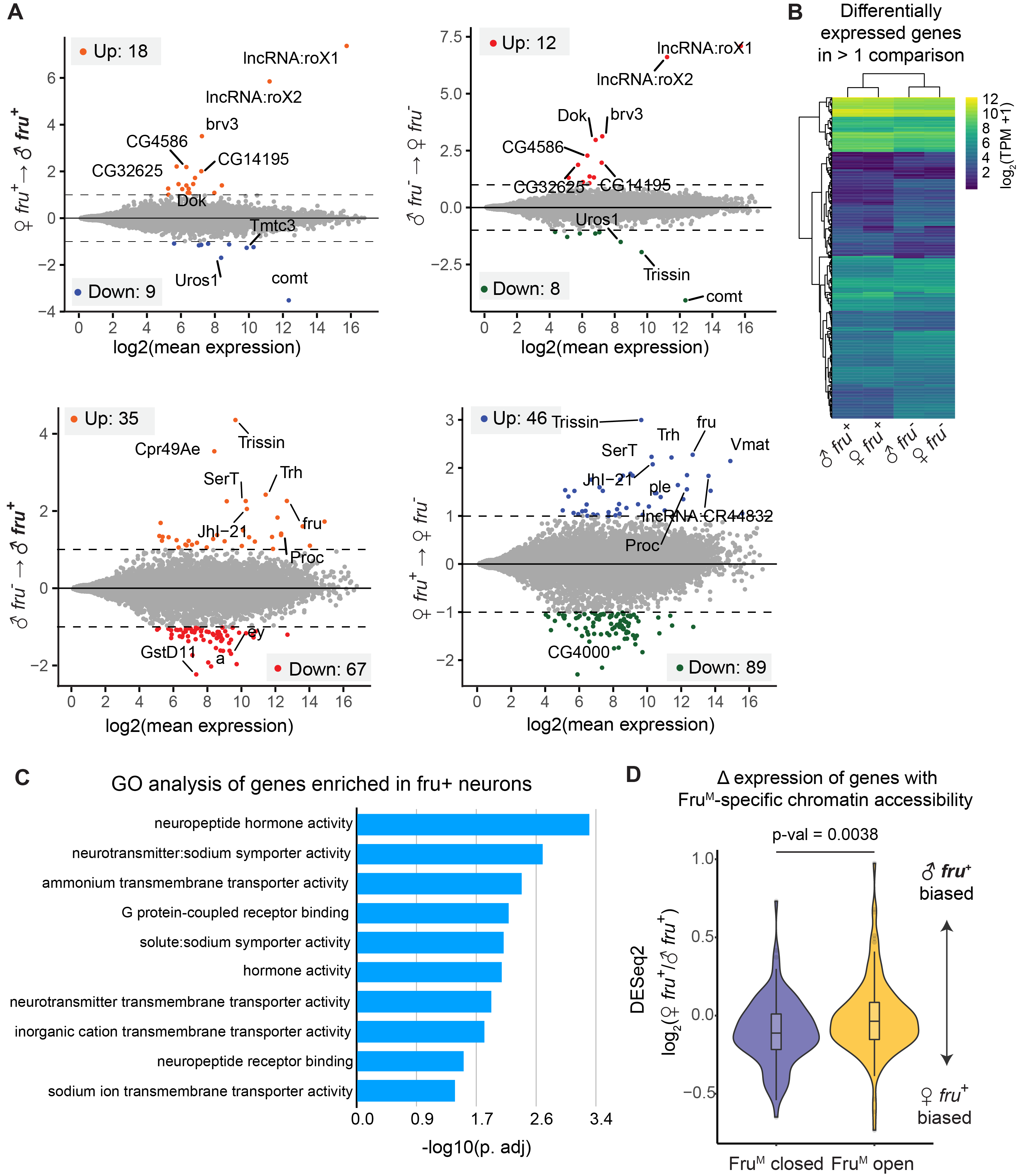

Supplement: S6 Fig — A. MA plots of differential RNA expression analysis between four datasets. B. Clustered heatmap of TPM values of genes with differential expression in one or more comparisons. Differential expression is dominated by fru status. C. Gene ontology analysis of genes enriched in both fru+ datasets (male and female) over fru- datasets. Enrichment is over a custom background of genes with expression > 10 TPM across the four datasets. D. Expression of 303 genes near FruM-closed or FruM-open ATAC-seq peaks in male versus female fru+ cells. P-value by Welch two-sample t-test. (TIF) [file pgen.1009338.s006.tif]

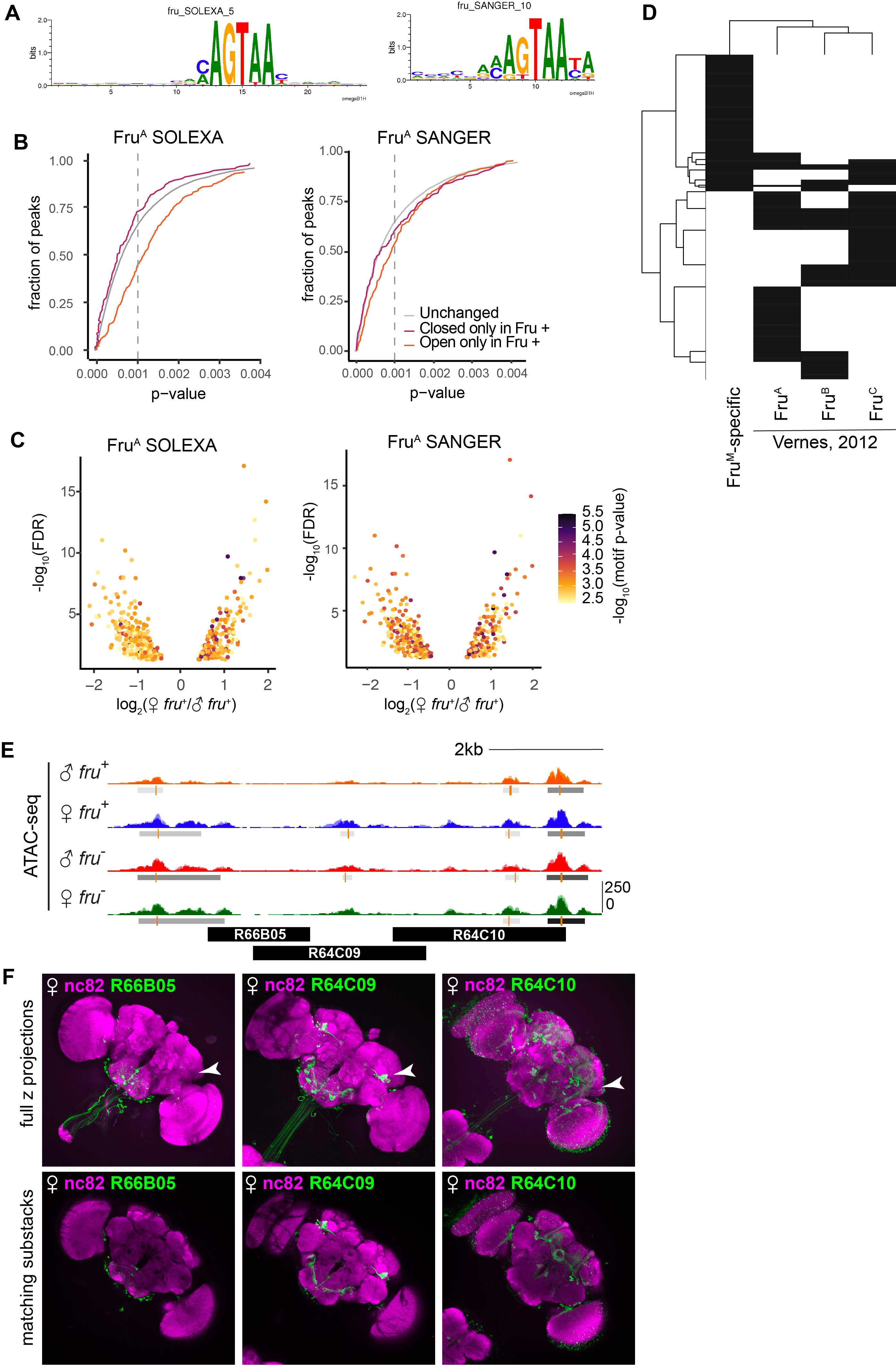

Supplement: S7 Fig — A. SELEX and SANGER FruA motifs. B. Cumulative frequency plots for SELEX and SANGER Fru (FruA) motifs (FlyFactorSurvey) across FruM-open, FruM-closed and unchanged regions. C. Volcano plots showing motif strengths across FruM-specific regions. D. Binary heat map of overlap of FruM-specific regions with previously reported FruM targets in S2 cells [66]. E. Expanded UCSC genome browser screenshot of ATAC-seq signal across a FruM-closed region covered by the enhancer reporter element R64C09 and two flanking regions R66B05 and R64C10. F. Adult female brain expression pattern of R66B05, R64C09, and R64C10 (green) driving expression with nc82 counterstain (magenta). Arrows point to region of neurons which show sexually dimorphic enhancer activity in R64C09. Images from Janelia FlyLight database. (TIF) [file pgen.1009338.s007.tif]
